# Genomic findings in non-cryptogenic cerebral palsy: a systematic review and meta-analysis

**DOI:** 10.3389/fneur.2026.1871290

**Published:** 2026-07-16

**Authors:** Paloma Arana-Rivera, Myriam Martín-Bermejo, Diana Marcela Nova-Díaz, Raquel Bernadó-Fonz, Nerea Gorría-Redondo, Diego Rivera, Laiene Olabarrieta-Landa, Sergio Aguilera-Albesa

**Affiliations:** 1 Pediatric Neurology Unit, Department of Pediatrics, Hospital Universitario de Navarra, Pamplona, Spain; 2 Departamento de Ciencias de la Salud, Universidad Pública de Navarra (UPNA), Pamplona, Spain; 3 Navarrabiomed–Complejo Hospitalario de Navarra–Universidad Pública de Navarra. (UPNA), Campus de Ciencias de la Salud, Pamplona, Spain; 4 Departamento de Económicas, Universidad Pública de Navarra (UPNA), Pamplona, Spain; 5 Instituto de Investigación Sanitaria de Navarra (IdiSNA), Pamplona, Spain

**Keywords:** cerebral palsy, exome sequencing, genetic testing, non-cryptogenic cerebral palsy, perinatal risk factors

## Abstract

**Background:**

The contribution of genomic variants to non-cryptogenic cerebral palsy (CP), defined by identifiable perinatal or acquired risk factors, remains incompletely characterized. This study aimed to estimate the frequency of reported pathogenic or likely pathogenic (P/LP) genomic findings in non-cryptogenic CP and compare it with cryptogenic cohorts.

**Methods:**

We conducted a systematic review and meta-analysis searching PubMed and Scopus to May 30, 2026, for sequencing-based CP studies with extractable non-cryptogenic data. Eligible approaches included whole-exome sequencing, whole-genome sequencing, and targeted next-generation sequencing panels. Risk of bias was assessed using an adapted JBI prevalence checklist. Pooled frequencies were calculated using random-effects models with logit transformation.

**Results:**

Eleven studies were included in the qualitative synthesis, and eight contributed to the meta-analysis. The primary non-cryptogenic analysis included 1,885 individuals, of whom 325 had reported P/LP genomic findings. The pooled frequency was 12.6% (95% CI 8.9–17.6; *I*^2^ = 80.6%), approximately one in eight tested individuals. In seven studies with cryptogenic subgroup data, the pooled frequency was 32.3% (95% CI 21.0–46.1; *I*^2^ = 69.9%). Cryptogenic cases were more than twice as likely to have a reported P/LP genomic finding as non-cryptogenic cases (risk ratio 2.21, 95% CI 1.56–3.14). Across all analyzed cohorts, the overall pooled frequency was 19% (95% CI 12–28). Prematurity was generally associated with lower frequencies, whereas selected hemorrhagic or cerebrovascular phenotypes showed enrichment for *COL4A1/COL4A2-*related findings.

**Interpretation:**

Reported P/LP genomic findings occur in a clinically meaningful subset of individuals with non-cryptogenic CP, although less frequently than in cryptogenic CP. Interpretation is limited by heterogeneous definitions of non-cryptogenic CP and incomplete genotype–phenotype adjudication across studies. These findings support careful assessment of perinatal risk factors, neuroimaging patterns, and genotype–phenotype concordance when interpreting genomic results.

**Systematic review registration:**

CRD420251169588, https://www.crd.york.ac.uk/PROSPERO/view/CRD420251169588.

## 1 Introduction

Cerebral palsy (CP) is the most common cause of motor disability in childhood. It is defined as an early-onset, lifelong neurodevelopmental condition that limits activity because of impaired development of movement and posture (1). Its prevalence is about 1.6 cases per 1,000 live births (2) and arises from diverse etiologies; the most recognized are prematurity, hypoxic–ischemic injury, and perinatal stroke (3–5), usually classified as non-cryptogenic. In some children, however, no clear cause can be identified, leading to the description of idiopathic or cryptogenic CP.

This heterogeneous origin has challenged efforts to concisely define CP for more than 150 years, and in the genomic era this complexity has become even more apparent. Advances in genomic sequencing have revealed that a subset of children, previously labeled as having cryptogenic CP, harbor single-gene disorders whose early clinical manifestations are indistinguishable from those of more traditional forms of CP (6, 7). Molecular analyses have also shown that, even in non-cryptogenic cases, the sentinel environmental event may be secondary to an underlying genetic vulnerability (8). Thus, genomic technologies have opened new avenues in the study of CP.

In particular, the introduction of whole-exome sequencing (WES), whole-genome sequencing (WGS) and clinical exome sequencing (CES) has enabled more accurate estimates of the proportion of patients with underlying genetic findings. Reported frequencies of pathogenic or likely pathogenic (P/LP) findings in CP vary widely across studies. Atypical CP presentations, often enriched for genetic etiologies, show the highest rates, approaching 50% (9–11). When cryptogenic and non-cryptogenic CP are analyzed separately, a prior meta-analysis reported proportions of P/LP findings of 35% (95% CI 27–45%) and 7% (95% CI 4–12%) respectively (12). Together, these findings support the view that cryptogenic CP carries a higher genetic burden and have contributed to the prevailing perception that non-cryptogenic CP is largely environmental. Nevertheless, previous studies were not designed to specifically examine non-cryptogenic cohorts in depth. In practice, non-cryptogenic cases are included in genetic studies at a frequency approximately 10 times lower than cryptogenic cases (13). To address this gap, we undertook a systematic review and meta-analysis to quantify the frequency of reported P/LP genomic variants in non-cryptogenic CP. Our aims were to provide pooled estimates, compare these findings with cryptogenic cohorts, explore sources of heterogeneity, and identify recurrently reported genes.

## 2 Methods

### 2.1 Study design and reporting guidelines

Our primary objective was to estimate the frequency of reported P/LP genomic variants in non-cryptogenic CP. Secondary aims were to compare these findings between non-cryptogenic and cryptogenic cohorts, when available, and to identify recurrently reported genes. The review was conducted according to PRISMA 2020 guidelines (Preferred Reporting Items for Systematic Reviews and Meta-Analyses) (14). The corresponding checklist is provided in Supplementary Data S1. The protocol for this systematic review was registered in PROSPERO (registration number: CRD420251169588).

### 2.2 Eligibility criteria

Original human research studies were included if they: (1) investigated cohorts of individuals with non-cryptogenic CP or reported an identifiable non-cryptogenic CP subgroup; (2) applied genomic sequencing approaches, including WES, WGS, CES or diagnostic NGS panels; and (3) included at least 10 participants.

Studies were excluded if they: (1) did not provide an identifiable non-cryptogenic CP subgroup; (2) did not include genomic testing; (3) were restricted to copy number variant (CNV) analyses, chromosomal microarray, candidate-gene testing, or association analyses without patient-level clinically diagnostic or contributory P/LP findings; (4) were not original research articles; (5) were restricted to single-gene analyses; or (6) were written in a language other than English or Spanish.

### 2.3 Operational definitions

#### 2.3.1 Non-cryptogenic cohort

CP with at least one identifiable antenatal, perinatal, or postnatal risk factor considered by the original study authors to provide a plausible explanation for the CP phenotype. This included, among others, prematurity, perinatal stroke, hypoxic–ischemic encephalopathy, kernicterus, infection, or cardiorespiratory arrest (3). Congenital brain malformations were not considered non-cryptogenic risk factors, as they reflect disruptions in early brain development rather than acquired antenatal, perinatal, or postnatal events (15).

#### 2.3.2 Cryptogenic cohort

CP without identifiable risk factors, or CP in which the available factors were insufficient to explain the clinical and neuroimaging findings. Cohorts labeled as cryptogenic, unexplained, or idiopathic may include heterogeneous cases, including atypical CP presentations, developmental regression, progressive symptoms, or features suggestive of a non-static course or alternative etiology.

Study-level classification decisions and handling of atypical, CP-like, or diagnostic-interface cases are summarized in Supplementary Data S2.

### 2.4 Search strategy

We systematically searched PubMed and Scopus up to May 30, 2026, using database-specific strategies. PubMed combined cerebral palsy, genomic testing, and environmental or acquired risk-factor terms. Scopus used a broader cerebral palsy and genomic testing strategy, excluding reviews, to maximize sensitivity for studies with extractable non-cryptogenic subgroup data. Duplicates were removed using Zotero (version 7.0.29) and Rayyan (16). Two reviewers independently screened records in two phases: title/abstract screening and full-text review. Discrepancies were resolved by consultation with a third independent reviewer. The complete search strategy and full-text exclusions with reasons are documented in Supplementary Data S3.

### 2.5 Risk of bias assessment

Risk of bias was assessed using an adapted version of the JBI Critical Appraisal Checklist for Studies Reporting Prevalence Data (17), tailored to studies reporting cohort-level proportions of individuals with CP carrying reported P/LP genomic variants. Because this outcome is expressed as a proportion among tested individuals, the JBI prevalence framework was adapted to assess sampling, case ascertainment, outcome definition, and reporting. The adapted domains and rating criteria are provided in Supplementary Data S4.

We additionally assessed phenotype-level robustness using the six study-level criteria proposed by Wilson et al. (18) for genetic studies in CP. For studies in which classification according to these criteria had already been reported, we retained the published classification. Studies without a prior classification were independently assessed in the present review using the same criteria. This Wilson-informed assessment was used to inform applicability and causal interpretation, not as a replacement for the JBI appraisal or as a weighting factor in the meta-analysis. The detailed assessment is provided in Supplementary Data S5.

### 2.6 Data extraction

For genomic testing, we recorded the number of individuals tested, the testing technique, including WES, WGS, CES, or diagnostic NGS panels, and the number of individuals with reported P/LP genomic findings counted by the original study authors in the final positive genetic findings for each cohort or subgroup. We also recorded whether CNVs and variants of uncertain significance (VUS) were reported by the original study, and whether CNVs were included in the reported P/LP findings.

The primary outcome was the frequency of reported P/LP genomic findings in non-cryptogenic CP cohorts or subgroups. This outcome could include single-nucleotide variants, small insertions/deletions, and CNVs when these were included as part of the sequencing-based findings reported by the original study.

Reported P/LP genomic findings were not automatically considered causative or diagnostic by this review. When available, we extracted how the original study authors interpreted these findings, including whether they were considered diagnostic, probably diagnostic, contributory, risk-related, or of uncertain relationship to the CP phenotype. Most included studies explicitly applied ACMG or ACMG/AMP criteria (19). In the remaining studies, pathogenicity classifications were accepted as reported by the original authors, as variant-level data were not consistently available to allow independent reassessment.

Additional variables included whether results were reported separately for non-cryptogenic and cryptogenic CP when both groups were available, whether genetic findings were analyzed in relation to specific perinatal factors, recurrently implicated genes, and whether each study was eligible for qualitative synthesis only or for quantitative meta-analysis. Data extraction was performed independently by two reviewers. Discrepancies were resolved by consultation with a third independent reviewer.

Studies retained for qualitative synthesis only were described narratively and were not included in pooled estimates when they lacked an extractable patient-level P/LP genomic finding frequency for the non-cryptogenic group.

### 2.7 Data synthesis and statistical analysis

The primary meta-analysis estimated the pooled proportion of individuals with reported P/LP genomic findings in studies or subgroups with extractable non-cryptogenic CP data according to the operational definitions described above. Random-effects meta-analyses of proportions were performed using a logit transformation and Hartung-Knapp adjustment. Between-study heterogeneity was assessed using Cochran’s Q statistic, τ^2^, and *I*^2^.

Cryptogenic cohorts were analyzed only as secondary comparators when extractable data were available from the included studies. Subgroup meta-analysis was used to compare the proportion of individuals with reported P/LP genomic findings between non-cryptogenic and cryptogenic cohorts, with differences assessed using the χ^2^ test for subgroup differences. For studies reporting both non-cryptogenic and cryptogenic subgroups, direct study-level comparisons were summarized using risk ratios, with risk differences and number needed to test reported as complementary measures.

Overall cohort-level proportions of reported P/LP genomic findings were summarized as a contextual analysis using the total CP cohort reported in each study. This analysis could include participants who were not assignable to the non-cryptogenic or cryptogenic subgroups based on the published data.

Sensitivity analyses included exclusion of small studies or subgroups, leave-one-out procedures, and Baujat plots to identify studies contributing disproportionately to heterogeneity. Additional robustness analyses were performed by excluding influential studies identified in sensitivity analyses. Potential publication bias was assessed by visual inspection of funnel plots; these analyses were considered exploratory given the limited number of included studies. The certainty of evidence was not formally graded because the included studies were observational, heterogeneous in design and reporting, and the review focused on pooled proportions rather than intervention effects. All analyses were performed in R version 4.4.3 using the *meta* package, within RStudio version 2025.05.0 + 496 (20, 21).

### 2.8 Ethical approval

This meta-analysis uses only published, de-identified data; therefore, ethical approval was not applicable.

## 3 Results

### 3.1 Search results

The database searches retrieved 1,369 records: 682 from Scopus and 687 from PubMed. After removing 207 duplicates, 1,162 records were screened by title and abstract, leading to the exclusion of 1,126 articles.

**Figure 1:**
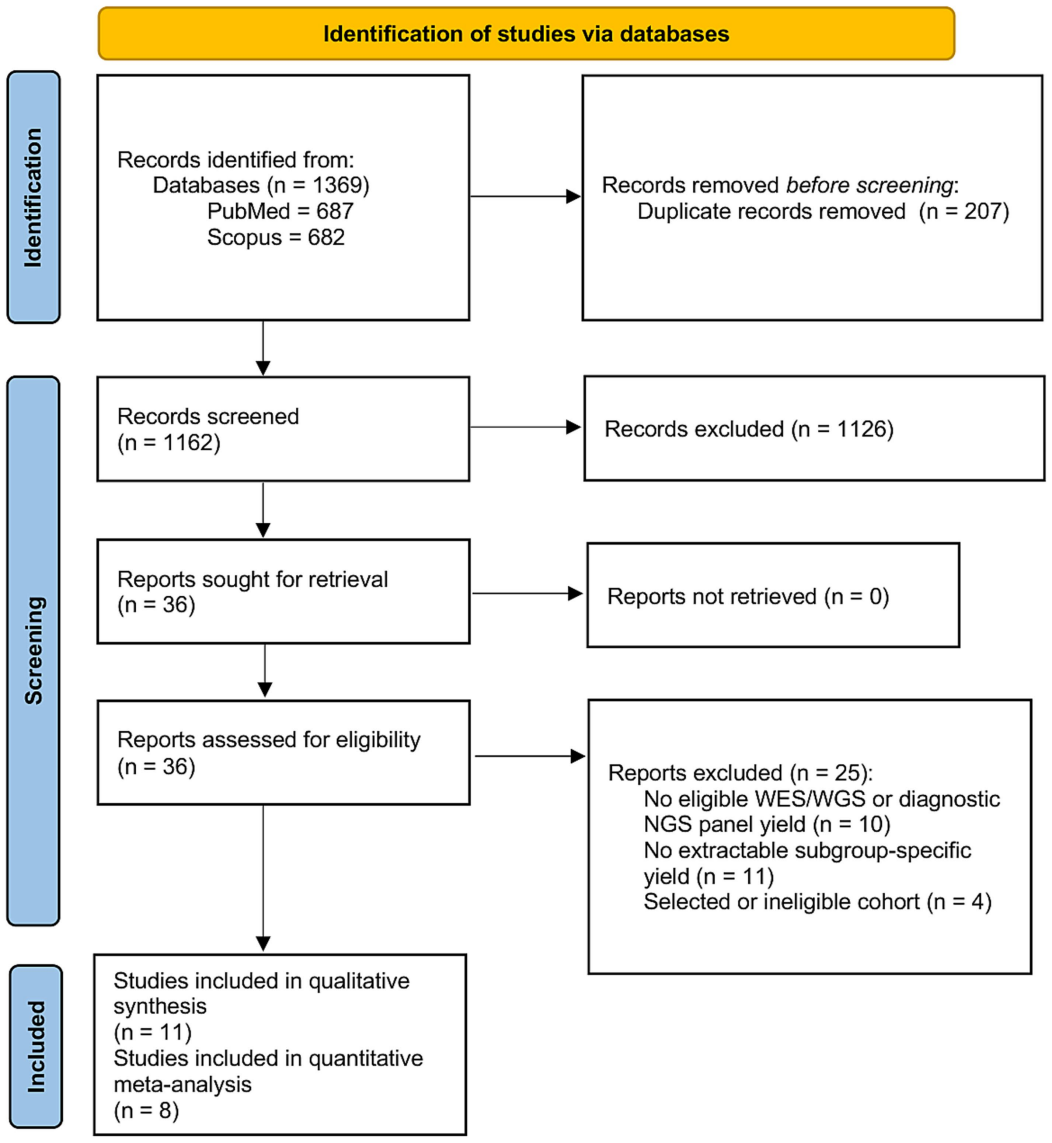
PRISMA 2020 flow diagram of study selection. A total of 1,369 records were retrieved through database searches, including 687 from PubMed and 682 from Scopus. After removal of 207 duplicate records, 1,162 records were screened by title and abstract, and 1,126 were excluded. Thirty-six full-text articles were assessed for eligibility, of which 25 were excluded for predefined reasons: no eligible WES/WGS or diagnostic NGS panel yield (*n* = 10), no extractable subgroup-specific yield (*n* = 11), or selected or ineligible cohort (*n* = 4). Ultimately, 11 studies were included in the qualitative synthesis, and eight studies were included in the quantitative meta-analysis. CP, cerebral palsy; NGS, next-generation sequencing; WES, whole-exome sequencing; WGS, whole-genome sequencing.

Thirty-six full-text articles were assessed for eligibility. Of these, 25 were excluded for the following reasons: no eligible WES, WGS, CES or diagnostic NGS panel-based yield was reported (*n* = 10), no extractable subgroup-specific yield was available (*n* = 11), or the cohort was selected or otherwise ineligible (*n* = 4). Ultimately, 11 studies were included in the qualitative synthesis, of which eight provided extractable subgroup-specific data and were included in the quantitative meta-analysis. The study selection process is summarized in Figure 1. Full-text articles assessed for eligibility, reasons for exclusion, and study-level inclusion decisions are detailed in Supplementary Data S3.

### 3.2 Study selection and characteristics

Eleven studies were included in the qualitative synthesis. Eight studies published between 2020 and 2026 provided extractable data for the primary quantitative analysis of reported P/LP genomic findings in non-cryptogenic CP (22–29), comprising 2,625 individuals with CP as defined by the source studies and including cohorts from North America, Europe, Asia, and Australia. Seven of these studies also reported cryptogenic subgroup data and were included in paired comparative analyses between cryptogenic and non-cryptogenic CP. Thys et al. (29) contributed only to the non-cryptogenic analysis because it focused specifically on CP following perinatal cerebrovascular events. The main characteristics of the included studies, including sequencing method, cohort size, CP subgroup classification, and contribution to the quantitative synthesis, are summarized in Table 1.

**Table 1:** Characteristics of studies included in the quantitative synthesis.

Study	Sequencing method	Variant types analyzed	Sample size	Overall reported P/LP findings	Non-cryptogenic cohort, *n*	P/LP findings in non-cryptogenic cohort	Cryptogenic cohort, *n*	P/LP findings in cryptogenic cohort
May et al. (22)	WES	SNVs, indels	151	9.3% (14/151)	123	8.1% (10/123)	28	14.3% (4/28)
Chopra et al. (23)	WES	SNVs, indels	44	22.7% (10/44)	20	15.0% (3/20)	24	29.2% (7/24)
Mei et al. (24)	CES/WES	SNVs, CNVs, indels	217	35.9% (78/217)	85	16.5% (14/85)	114	48.2% (55/114)
Pavelekova et al. (25)	WES	SNVs, indels	70	27.1% (19/70)	36	13.9% (5/36)	31	45.2% (14/31)
Wang et al. (26)	WES	SNVs, CNVs	1,578	24.5% (387/1,578)	1,175	21.2% (249/1,175)	403	34.2% (138/403)
Fehlings et al. (27)	WGS	SNVs, indels, CNVs, repeat expansions, mtDNA variants	327	11.3% (37/327)	289	11.1% (32/289)	23	13.0% (3/23)
Almansa et al. (28)	WES	SNVs, indels	177	18.1% (32/177)	96	7.3% (7/96)	81	30.9% (25/81)
Thys et al. (29)	WES	SNVs, CNVs	61	8.2% (5/61)	61	8.2% (5/61)	NA	NA

P/LP, pathogenic or likely pathogenic; WES, whole-exome sequencing; WGS, whole-genome sequencing; CES, clinical exome sequencing; SNV, single-nucleotide variant; CNV, copy number variant; mtDNA, mitochondrial DNA; NA, not available.

Three studies, Jin et al. (30), van Eyk et al. (31), and Yi et al. (32), were retained for qualitative synthesis only. These studies were informative for genetic contributions in environmental, vascular, or clinically selected CP contexts with specific non-cryptogenic CP information, but they did not provide subgroup-specific patient-level data compatible with quantitative pooling of reported P/LP genomic findings.

Definitions of cryptogenic and non-cryptogenic CP (33, 34), handling of mimics and VUS, and genetic testing strategies varied across studies. Most quantitatively included studies used exome sequencing, whereas Fehlings et al. (27) used genome sequencing. Detailed study characteristics are provided in Supplementary Data S2.

### 3.3 Methodological quality, risk-of-bias and phenotype-confidence assessment

Among the eight studies included in the quantitative meta-analysis (22–29), overall risk of bias was rated as moderate. The main recurrent limitations were referral-based or tertiary-center recruitment, small subgroup sizes in some studies, heterogeneous definitions of cryptogenic and non-cryptogenic CP, variable handling of CP mimics, and differences in genetic testing strategies and variant adjudication.

The studies retained for qualitative synthesis only also had relevant methodological or applicability limitations. These included indirectness of the reported genetic outcome, absence of extractable subgroup-specific patient-level counts of reported P/LP genomic findings, use of predicted damaging variants or burden/enrichment analyses, and restriction of genetic testing to clinically selected subsets.

Overall, the risk-of-bias assessment supported cautious quantitative synthesis of the eight eligible studies and narrative interpretation of the remaining studies. Full domain-level judgments and study-specific rationales are provided in Supplementary Data S4.

The phenotype-confidence assessment revealed variability in the completeness and quality of CP phenotypic reporting across the included studies (18). Overall, this assessment highlighted that, although most studies provided a standard CP definition and motor phenotype description, fewer reported systematic diagnostic reconfirmation, confirmation at or after 4 years of age, individual-level phenotypic data, or explicit exclusion of progressive or non-static CP mimics. The detailed assessment is provided in Supplementary Data S5.

### 3.4 Reported P/LP genomic findings in non-cryptogenic CP

**Figure 2:**
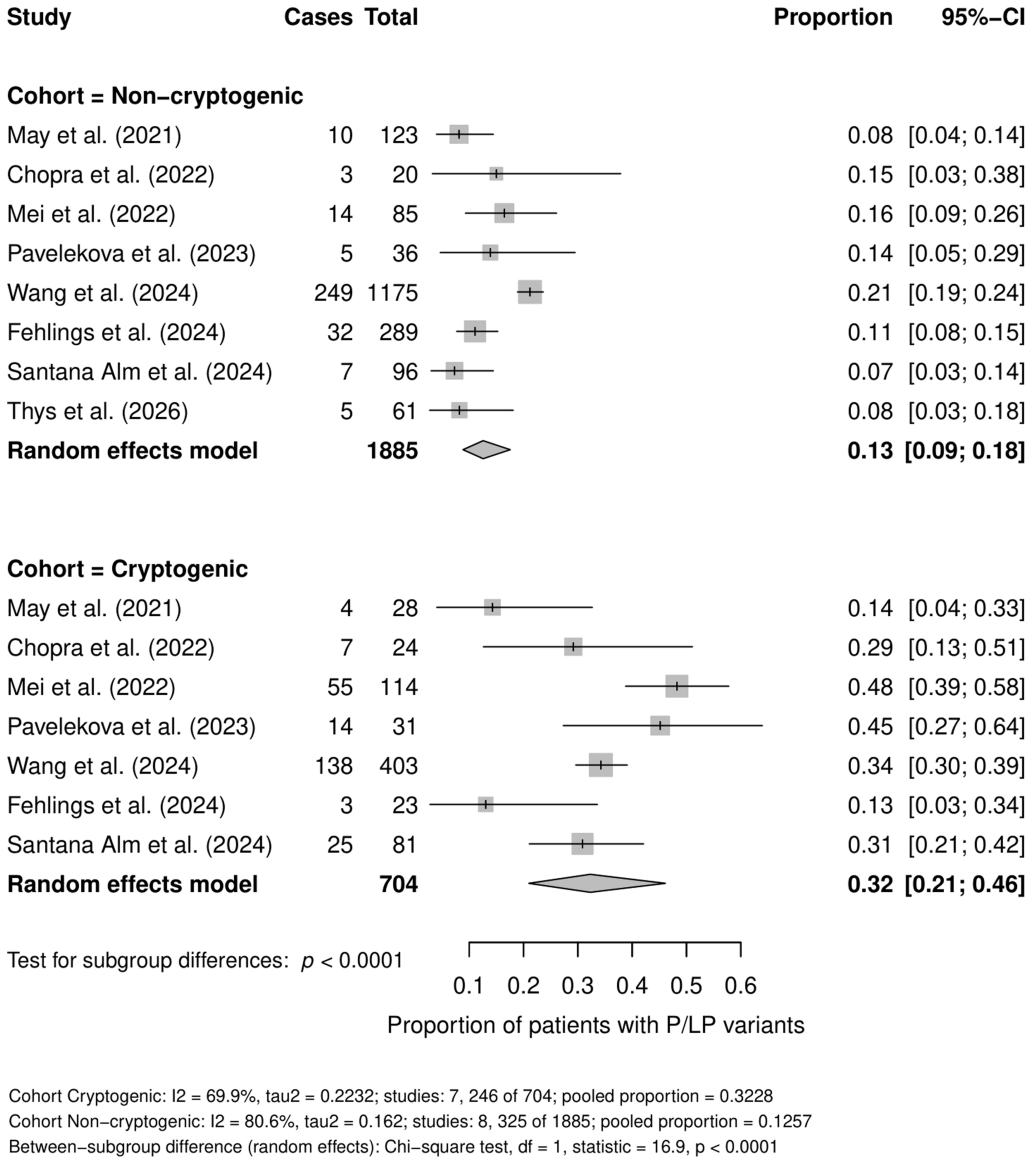
Forest plot comparing the pooled proportions of reported P/LP genomic findings in non-cryptogenic and cryptogenic cerebral palsy cohorts. Squares represent study-specific proportions, with horizontal lines indicating 95% confidence intervals, and diamonds represent pooled estimates calculated using a random-effects model. The pooled proportion was 12.6% (95% CI, 8.9–17.6) in non-cryptogenic cerebral palsy and 32.3% (95% CI, 21.0–46.1) in cryptogenic cerebral palsy. The difference between cohorts was statistically significant (*p* < 0.0001). CI, confidence interval; P/LP, pathogenic or likely pathogenic.

The primary non-cryptogenic analysis included eight studies (22–29) comprising 1,885 individuals classified as having non-cryptogenic CP. Overall, 325 individuals had reported P/LP genomic findings. The crude proportion was 17.2%, whereas the random-effects pooled frequency was 12.6% (95% CI 8.9–17.6; *I*^2^ = 80.6%, τ^2^ = 0.162) (Figure 2).

### 3.5 Comparison between cryptogenic and non-cryptogenic CP

Seven studies provided paired cryptogenic and non-cryptogenic subgroup data (22–28). These studies included 704 individuals with cryptogenic CP, of whom 246 had reported P/LP genomic findings. The crude frequency was 34.9%, whereas the random-effects pooled frequency was 32.3% (95% CI 21.0–46.1; *I*^2^ = 69.9%, τ^2^ = 0.2232) (Figure 2). This frequency was significantly higher than that observed in the corresponding non-cryptogenic subgroups (between-subgroup χ^2^ = 16.9, df = 1, *p* < 0.0001). In the paired comparative random-effects meta-analysis, cryptogenic cases were more than twice as likely to have a reported P/LP genomic finding as non-cryptogenic cases (RR 2.21, 95% CI 1.56–3.14; *I*^2^ = 48.7%). The pooled absolute risk difference was 17.1% (95% CI 8.5–25.7), corresponding to approximately one additional reported P/LP genomic finding for every six individuals tested in the cryptogenic rather than the non-cryptogenic subgroup.

### 3.6 Overall frequency of reported P/LP genomic findings

**Figure 3:**
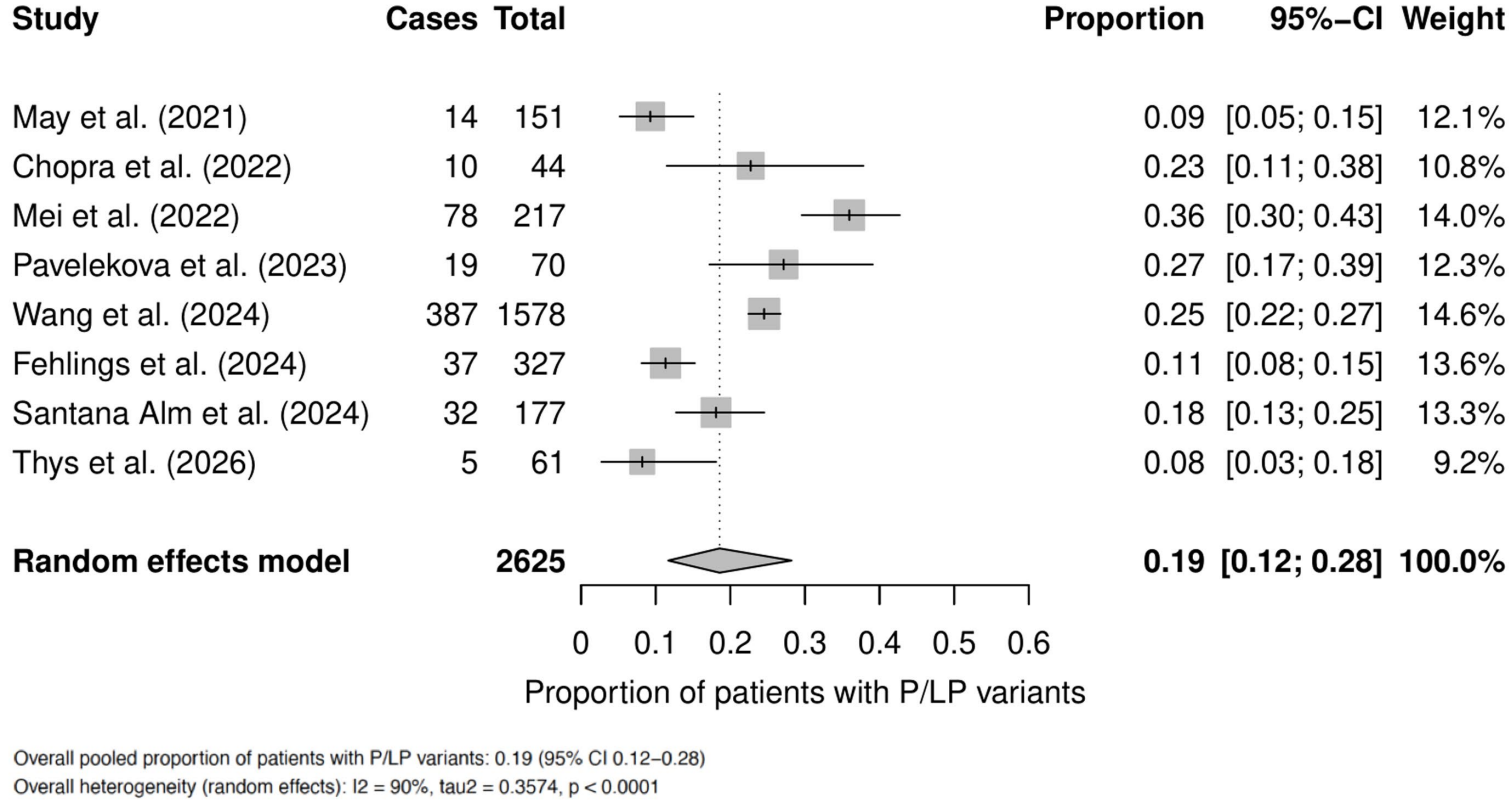
Forest plot showing the pooled proportion of reported P/LP genomic variants in the overall cerebral palsy cohort. Squares represent study-specific proportions, with horizontal lines indicating 95% confidence intervals, and the diamond represents the pooled estimate derived from a random-effects model. Across eight studies, the pooled proportion was 19% (95% CI, 12–28), with substantial between-study heterogeneity. CI, confidence interval; P/LP, pathogenic or likely pathogenic.

Across the eight studies included in the global quantitative analysis (22–29), 582 of 2,625 individuals had reported P/LP genomic findings. This global analysis used the total cohort-level numerator and denominator reported by each study, and therefore included all participants contributing to the quantitative synthesis, including individuals not assigned to the cryptogenic or non-cryptogenic comparative subgroups when applicable. The random-effects pooled proportion was 19% (95% CI 12–28), with substantial between-study heterogeneity (*I*^2^ = 90%, τ^2^ = 0.3574, *p* < 0.0001) (Figure 3).

### 3.7 Heterogeneity and sensitivity analyses

Sensitivity analyses supported the robustness of the non-cryptogenic pooled estimate. In leave-one-out analyses, the pooled frequency remained between 11.0 and 13.5% after omitting individual studies. Wang et al. (26) was the main contributor to heterogeneity: its exclusion reduced *I*^2^ from 80.6 to 0.3%, while the pooled frequency changed only modestly from 12.6 to 11.0% (95% CI 8.4–14.4).

A sensitivity analysis excluding Chopra et al. (23) was performed because of possible partial cohort overlap with Almansa et al. (28). Its exclusion had minimal impact on the pooled estimate, which remained 12.4% (95% CI 8.3–18.1).

The difference between cryptogenic and non-cryptogenic CP remained statistically significant after these sensitivity analyses. Full results are provided in Supplementary Data S6.

### 3.8 Publication bias

Assessment of publication bias was exploratory because of the small number of studies included in each analysis and the substantial between-study heterogeneity. Visual inspection of funnel plots for the global, non-cryptogenic, and cryptogenic analyses did not show a clear pattern of small-study effects. However, formal inference was limited, and publication bias could not be reliably excluded.

### 3.9 Risk-factor-specific patterns in non-cryptogenic cerebral palsy

Several studies investigated whether specific perinatal or neonatal risk contexts modified the frequency and interpretation of reported P/LP genomic findings. However, differences in how these factors were defined, recorded, and incorporated into subgroup analyses limited direct comparability across cohorts and precluded formal quantitative pooling by individual risk factor.

Prematurity was generally associated with a lower frequency of P/LP genomic findings compared with term birth (22, 23, 25–27). In contrast, one large study reported a higher frequency of P/LP genomic findings among individuals with perinatal asphyxia (26); however, this association was not consistently replicated across other studies.

Cerebrovascular and hemorrhagic presentations showed heterogeneous results. Reported P/LP genomic findings were uncommon across broad cerebrovascular-injury groups in some studies (22, 26). However, in the dedicated cohort of CP following perinatal cerebrovascular events, hemorrhagic presentations showed a higher frequency of P/LP genomic findings than ischemic stroke, largely driven by *COL4A1* variants (29). No consistent association emerged for other reported risk factors, including sepsis or kernicterus.

Overall, the descriptive synthesis indicated that reported P/LP genomic findings in non-cryptogenic CP are not uniformly distributed across perinatal risk contexts, but available data were insufficient for risk-factor-specific estimates.

### 3.10 Gene-level findings and clinical adjudication

Gene-level analyses were restricted to reported P/LP genomic findings with extractable gene-level data. Genes derived exclusively from qualitative-only burden or enrichment analyses were not included in quantitative gene counts.

Across the included studies with available gene-level data, reported findings were highly heterogeneous, with most recurrent genes identified in only small numbers of individuals. The most recurrent findings involved *COL4A1* and *COL4A2* with 12 and 4 individuals, respectively (23, 26–29, 31), consistent with the contribution of type IV collagen-related small-vessel disease, particularly in vascular or hemorrhagic presentations. Other recurrent genes were reported in small numbers of patients and mapped to heterogeneous biological categories, including coagulation and thrombophilia pathways, cytoskeletal organization and neuritogenesis pathways, and genes classically associated with neurogenetic disorders that may resemble or overlap with CP, such as hereditary spastic paraplegia, epileptic encephalopathies, and broader neurodevelopmental disorders.

Source-level interpretation of reported P/LP findings varied across studies. Although included studies generally applied variant classification and some level of clinical interpretation, the published reporting did not allow a uniform case-level distinction between variants considered direct contributors to the CP phenotype, modifiers of disease risk or susceptibility, clinically relevant co-occurring diagnoses, or findings of uncertain contribution. This variability limited causal interpretation across studies and should be considered when comparing reported diagnostic yields. Source-level details for recurrent and extractable gene-level findings are provided in Supplementary Data S7.

## 4 Discussion

This systematic review and meta-analysis provides a specific estimate of the frequency of reported P/LP genomic findings in non-cryptogenic CP. The pooled proportion in non-cryptogenic CP was 12.6%, corresponding to approximately one in eight individuals tested. Although this frequency was lower than that observed in cryptogenic CP, where the pooled proportion was 32.3%, it remains clinically meaningful and falls within a range in which genomic testing is commonly considered in other neurodevelopmental disorders (35–37).

Compared with previous meta-analyses reporting yields of around 30% in overall CP and up to 50% in cryptogenic cohorts (5, 6, 10, 38–41), our overall estimate was lower, likely reflecting the specific focus on non-cryptogenic CP and the stricter separation of etiological subgroups. However, the frequency observed in non-cryptogenic CP was higher than earlier estimates of approximately 7% (12). These findings indicate that the presence of perinatal or neonatal risk factors reduces, but does not exclude, the probability of identifying relevant genomic findings. Therefore, non-cryptogenic CP should not be systematically excluded from genetic evaluation.

From a clinical perspective, these findings support a stratified rather than indiscriminate approach to genomic testing in non-cryptogenic CP. Stratification should consider both the type of acquired risk factor and how convincingly it accounts for the individual phenotype. Across the included studies, and in line with previous reports, prematurity was generally associated with a lower probability of identifying P/LP genomic findings (24, 26, 27, 42). This suggests that isolated prematurity, particularly when accompanied by a typical preterm-related brain injury pattern and a clinically concordant phenotype, may represent a lower-yield scenario. However, the presence of a recognized risk factor should not be interpreted in isolation. Genetic testing may still be relevant when the presumed acquired event is mild, poorly characterized, or leaves part of the clinical or neuroimaging phenotype unexplained. This is particularly important in vascular and hemorrhagic perinatal events. Although perinatal intracranial hemorrhage was associated with lower proportions of P/LP findings in some broader cohorts (22, 26), the dedicated cohort of CP following perinatal cerebrovascular injury showed that genetic findings were concentrated in hemorrhagic rather than ischemic presentations, particularly involving collagen-related genes, which are discussed below (29).

Throughout this review, we deliberately refer to P/LP genomic findings rather than confirmed genetic diagnoses. This distinction reflects the available evidence and avoids overinterpretation of reported findings. This is relevant because some variants reported in non-cryptogenic groups involve genes more commonly associated with broader neurodevelopmental or CP-like disorders than with acquired CP in the strict sense. For instance, the association reported by Wang et al. (26) between hypoxic–ischemic encephalopathy and variants in embryonic developmental pathways is difficult to interpret without detailed individual-level genotype–phenotype correlation. Such findings may reflect a direct genetic contribution, increased vulnerability to injury, phenotype overlap, or a co-occurring neurodevelopmental disorder. Therefore, the clinical value of genomic testing depends not only on detecting variants, but also on interpreting them within the clinical and neuroimaging context.

Among recurrent findings, *COL4A1* and *COL4A2* were the most consistently reported genes in non-cryptogenic cohorts. This is clinically relevant because type IV collagen-related disease has been associated with vascular brain injury phenotypes (43–46). Their recurrence suggests that collagen-related vasculopathy may contribute to a subset of CP cases with vascular or hemorrhagic presentations that might otherwise be interpreted as acquired. Identification of these variants may have implications for recurrence-risk counseling, family testing, and clinical follow-up. Accordingly, *COL4A1* and *COL4A2* should be considered in the genetic evaluation of children with antenatal or perinatal vascular or hemorrhagic events, particularly when the neuroimaging pattern or family history raises suspicion of an underlying vasculopathy.

CNVs and other structural genomic findings may also be relevant. Although CNV-focused studies did not meet the criteria for inclusion in the main quantitative estimate, they provide contextual evidence that structural genomic variation can contribute to CP phenotypes (6, 47). Future diagnostic frameworks should therefore integrate SNVs, indels, CNVs, and other variant classes within harmonized interpretation models.

This review has several limitations. Definitions of non-cryptogenic CP, phenotyping depth, neuroimaging classification, segregation data, and variant adjudication varied across studies, limiting causal interpretation and subgroup comparisons. Most studies also lacked harmonized denominators, severity grading of perinatal exposures, standardized assessment of clinical course, and neuroimaging-phenotype concordance, precluding formal meta-analysis by individual risk factor.

These issues are especially important when interpreting reported gene-level findings. P/LP variants in non-cryptogenic cohorts should be considered findings identified in children classified as non-cryptogenic by the original studies, rather than definitive evidence of causal genes for purely acquired CP. Broad definitions may inflate the apparent genomic yield if genetic CP, CP mimics, or diagnostic-interface cases are classified as non-cryptogenic because an incidental or insufficiently explanatory perinatal risk factor is present.

Future studies should improve clinical classification in CP by integrating the severity of perinatal risk factors, disease course, neuroimaging pattern, and genomic findings. This will be essential to determine whether identified variants represent a primary cause, a predisposition to injury, a modifier of outcome, or an unrelated finding. Prospective studies using standardized phenotyping, detailed neuroimaging, segregation analysis, and transparent genomic adjudication will help identify which children with non-cryptogenic CP benefit most from genetic testing.

## Data Availability

The original contributions presented in the study are included in the article/Supplementary material, further inquiries can be directed to the corresponding author.
